# Treatment Strategies for Peri-Implant Mucositis: The Final Stop for Preventing Peri-Implantitis

**DOI:** 10.1155/ijod/6901156

**Published:** 2025-04-28

**Authors:** Rafael Pereira, Hamoun Sabri, Paolo Nava, Abdusalam Alrmali, Hom-Lay Wang

**Affiliations:** Department of Periodontics and Oral Medicine, University of Michigan School of Dentistry, Ann Arbor, Michigan, USA

**Keywords:** clinical decision-making, decontamination, dental implants, inflammation, laser therapy, mucositis, oral hygiene, ozone, peri-implantitis, photochemotherapy, probiotics, risk factors, supportive therapy

## Abstract

**Objectives:** This article aims to provide a comprehensive overview of peri-implant mucositis, covering its etiology, risk factors, clinical features, diagnosis, and available treatment modalities.

**Methods:** A comprehensive electronic and manual search was performed on electronic databases. Studies with focus on peri-implant mucositis were included and reviewed comprehensively. Based on the latest evidence, decisional workflows and clinical recommendations were proposed.

**Results:** The review highlights the multifactorial etiology of peri-implant mucositis, primarily driven by bacterial biofilm accumulation. Key risk factors identified include poor oral hygiene, smoking, uncontrolled diabetes, and local factors such as implant surface characteristics and prosthesis design. Diagnostic criteria are based on the presence of clinical signs (inflammation, redness, swelling, and bleeding on probing (BoP)) and the absence of radiographic bone changes. Nonsurgical treatments, including mechanical debridement and photodynamic therapy (PDT), have shown effectiveness in managing the condition. However, the efficacy of adjunctive therapies remains inconclusive. Regular maintenance and optimal oral hygiene are critical in preventing and managing peri-implant mucositis.

**Conclusion:** Peri-implant mucositis remains a prevalent condition in implant dentistry, with nonsurgical treatment strategies showing promising outcomes in disease management. Further research is needed to establish the long-term effectiveness of adjunctive therapies and optimize preventive strategies for maintaining peri-implant health.

## 1. Background

The advancement of dental implants marks a significant breakthrough in modern dentistry, providing a reliable solution for tooth replacement and enhancing both function and esthetics. Despite their generally high success rates, dental implants are prone to various complications, including both mechanical and biological issues. Notably, peri-implant mucositis has emerged as a prominent concern in implant dentistry [1, 2].

Peri-implant mucositis is characterized by inflammation of the soft tissues surrounding dental implants, without accompanying pathologic bone loss. Its development is primarily attributed to the accumulation of plaque biofilm on the mucosal surfaces, akin to the etiology of gingivitis in natural teeth. This plaque-induced inflammation triggers an immune response, leading to tissue inflammation and potential complications [3].

Early diagnosis and effective management of peri-implant mucositis are decisive to prevent its progression to peri-implantitis, a more severe condition marked by bone loss beyond initial biological bone loss around dental implants. Furthermore, treating peri-implant mucositis is essential for maintaining a long-term success and stability of peri-implant soft and hard tissues.

This paper aims to provide a comprehensive overview of peri-implant mucositis, covering its etiology, risk factors, clinical features, diagnosis, and available treatment modalities. By enhancing our understanding of this condition and its management, dental clinicians can optimize patient care and ensure the longevity of dental implant restorations.

### 1.1. Peri-Implant Mucosa

The tissue surrounding dental implants is known as peri-implant mucosa. The characteristics of this mucosa are established during the healing process that occurs after implant placement. As the mucosa heals, it forms a soft tissue attachment (transmucosal attachment) to the implant. This attachment acts as soft tissue seal, preventing oral cavity products from reaching the bone tissue and ensuring the successful integration and stable fixation of the implant.

The peri-implant mucosa plays a critical role in preventing peri-implant mucositis, the precursor to peri-implantitis. This mucosa forms a protective barrier around dental implants, serving as the first line of defense against microbial invasion and plaque accumulation. When the peri-implant mucosa is healthy, it provides a resilient seal against bacterial infiltration, thereby minimizing the risk of inflammation and infection. Additionally, a healthy peri-implant mucosa promotes tissue stability and integration, contributing to the long-term success of dental implants. Proper oral hygiene practices, including regular brushing and flossing, along with professional maintenance care, are essential for preserving the integrity of the peri-implant mucosa and preventing the onset of peri-implant mucositis. By maintaining a healthy peri-implant mucosa, clinicians can diminish the risk of peri-implant mucositis development and ensure the longevity of implant-supported restorations.

Prior to the discussion on diseases, it is crucial to establish a clear definition of what constitutes peri-implant health. According to Araujo and Lindhe [3], a healthy peri-implant mucosa is composed of a central connective tissue core, which can be covered by either a keratinized or nonkeratinized epithelium. It is characterized by the absence of clinical signs of inflammation, such as redness, swelling, and bleeding upon probing.

The peri-implant mucosa and the gingiva share several clinical and histological characteristics, while they also have their differences. Studies in both humans and preclinical models have examined the response of the gingiva and peri-implant mucosa to early and prolonged periods of plaque formation. In both cases, the host response to bacterial challenge includes the development of clinical signs of inflammation and the formation of inflammatory lesions in the connective tissues of the mucosa or gingiva.

Plaque accumulation and the soft tissue response to microbial challenge, such as inflammation and changes in probing pocket depth (PPD), were found to occur in a similar manner in the tooth and implant regions of the oral cavity. The presence of plaque was associated with clinical signs of soft tissue inflammation and an increase in the infiltration of inflammatory cells in the tissues surrounding both teeth and implants.

### 1.2. Peri-Implant Diseases

Peri-implant diseases, such as peri-implant mucositis and peri-implantitis, were initially defined and described at the First European Workshop on Periodontology in 1993. More recently, during the World Workshop on Periodontal and Peri-implant Diseases and Conditions in 2017, official case definitions and diagnostic criteria for peri-implant health, peri-implant mucositis, and peri-implantitis were proposed [4].

In previous workshops, peri-implant mucositis has been described as an inflammatory condition affecting the mucosa surrounding dental implants, without any accompanying loss of supporting bone. The current criteria for defining peri-implant mucositis include the presence of inflammation in the peri-implant mucosa, as indicated by profuse bleeding on probing (BoP). Additional signs of this condition may include redness, swelling, and suppuration, as well as increased PPD compared to baseline (due to swelling or decrease in probing resistance) and absence of further bone loss following initial healing (<2 mm). Furthermore, it is important to note that there should be no ongoing marginal peri-implant bone loss for a diagnosis of peri-implant mucositis [4, 5].

While the main focus of this manuscript is not on peri-implantitis, it is essential to define this condition to comprehend its progression. Peri-implantitis, as described by Schwarz et al. [6], is characterized by inflammation in the connective tissue surrounding the implant and the gradual loss of supporting bone. The extent of the lesions in peri-implantitis exceeds those observed in peri-implant mucositis, reaching into the supracrestal connective tissue zone.

Clinical indicators of peri-implantitis include signs of inflammation, such as bleeding upon probing and/or suppuration, as well as increased PPDs and/or recession of the mucosal margin. In addition, radiographic evidence of bone loss compared to previous examinations is observed. In cases where previous examination data or radiographs are unavailable, the diagnosis may be based on bleeding (and/or suppuration) upon gentle probing, PPDs of ≥6 mm, and bone levels ≥3 mm apical to the most coronal portion of the intraosseous part of the implant [4].

By simplifying the definitions, gingivitis refers to inflammation of the gingiva without any loss of periodontal attachment, while peri-implant mucositis refers to inflammation of the peri-implant mucosa without ongoing marginal bone loss. Both conditions exhibit clinical signs of inflammation, including redness, swelling, and bleeding upon gentle probing. Suppuration is an additional sign observed in peri-implant mucositis. Importantly, both diseases can be reversed clinically by reinstituting plaque control. However, gingivitis typically resolves within 1 week, whereas peri-implant mucositis may take longer than 3 weeks to resolve.

### 1.3. Epidemiology

The evaluation of peri-implant diseases has predominantly been conducted through cross-sectional studies, providing insights into the prevalence of these conditions. However, there remains controversy surrounding the exact prevalence of peri-implant diseases.

Peri-implant mucositis has been reported to occur in approximately 80% of individuals with dental implants, while peri-implantitis affects a range of 28%–56% of subjects [7]. The variability in reported prevalence across different studies can be attributed, in part, to methodological differences, including the use of heterogeneous case definitions.

Reports on the prevalence of mucositis and peri-implantitis have shown a wide range, varying from 5% to 63.4% [8]. This significant range is primarily due to variations in study design, population sizes, risk profiles, and statistical approaches. According to Derks and Tomasi's [9] meta-analysis, the weighted mean prevalence of peri-implant mucositis was found to be 43% (ranging from 19% to 65%), while for peri-implantitis, it was 22% (ranging from 1% to 47%). They also noted that as the occurrence of peri-implant diseases accumulates over time, studies with longer follow-up periods are expected to report higher proportions of these diseases.

### 1.4. Etiology

According to Lindhe et al. [7], the etiology and pathogenesis of peri-implant diseases, including the transition from healthy peri-implant mucosa to peri-implant mucositis and from peri-implant mucositis to peri-implantitis, are similar to those observed in periodontal diseases affecting natural teeth.

Healthy peri-implant mucosa is characterized by a nonkeratinized oral epithelium barrier facing the implant or abutment surface. The presence of inflammatory cells forms a soft tissue seal that separates the peri-implant attachment from the oral cavity. Peri-implant mucositis develops from healthy peri-implant mucosa as a result of bacterial biofilm accumulation around dental implants. Studies in humans have demonstrated a cause-and-effect relationship between the experimental buildup of bacterial biofilms on titanium dental implants and the development of an inflammatory response. However, further evidence is needed to establish a definitive cause-and-effect relationship, particularly through proof of the reversibility of mucosal health to pre-inflammatory levels [5].

Salvi et al. [10] showed that gingivitis and peri-implant mucositis were reversible at the biomarker level. However, in clinical terms, it was found that 3 weeks of resumed plaque control did not fully restore the mucosal health to preexperimental levels, indicating that longer healing periods are required. In contrast, Meyer et al. [11] demonstrated the reversibility of experimentally induced peri-implant mucositis in an elderly patient sample (≥70 years) by showing that all clinical parameters assessed returned to pre-experimental levels after 3 weeks of reinstituted biofilm control.

Peri-implant sites affected by mucositis exhibit biofilms with lower complexity and anaerobic bacteria compared to dental sites with gingivitis in the same patient [12]. However, the peri-implant microbiota possesses unique characteristics, reflecting the environmental differences between dental implants and natural teeth. Peri-implant biofilms tend to have lower microbial diversity than subgingival biofilms, with a predominance of gram-negative anaerobes such as *Porphyromonas*, *Treponema*, and *Campylobacter* [13, 14]. Moreover, the anatomical and histological characteristics of peri-implant tissues, including the absence of the periodontal ligament and the circumferential arrangement of connective fibers, result in a less effective barrier to microbial invasion, predisposing peri-implant sites to dysbiosis [14, 15]. In the short term, adjunctive antimicrobial therapy combined with mechanical debridement can positively alter the composition of biofilms in both dental and peri-implant sites, promoting a more balanced and healthier subgingival environment [16].

According to Kwon et al. [17], the rescue and maintenance of dental implants affected by peri-implant diseases can be achieved through the targeted elimination of both nonsurgical and surgical etiologic factors. To ensure long-term success, proper peri-implant supportive therapy should be implemented. It is crucial to carefully plan and execute implant therapy, taking into account potential causes and contributing factors to prevent biological complications.

#### 1.4.1. Risk Indicators/Factors for Peri-Implant Mucositis

Risk factors are environmental, behavioral, or biological factors that have been confirmed through longitudinal studies. Their presence directly increases the likelihood of a disease occurring, while their absence or removal reduces that probability. On the other hand, risk indicators are associated with a disease based on cross-sectional or retrospective studies and have not been confirmed through longitudinal investigations.

Heitz-Mayfield and Salvi [5] provided an overview of systemic and local risk indicators for peri-implant mucositis, based on cross-sectional or retrospective study designs. Although there is limited information available on risk factors specifically for peri-implant mucositis, there are similarities with risk indicators for periodontal disease.

Systemic risk indicators for peri-implant mucositis include smoking, poorly controlled diabetes mellitus, radiation therapy, and age (Table 1). These factors have been associated with an increased risk of developing peri-implant mucositis.

As for local risk indicators, the author highlighted several factors, including poor oral hygiene, noncompliance and/or lack of supportive implant therapy, characteristics of implant materials and surfaces, prostheses design, dimensions of keratinized peri-implant mucosa, and the presence of excess cement (Table 1). These local factors can contribute to an increased risk of peri-implant mucositis.

**Figure 1:**
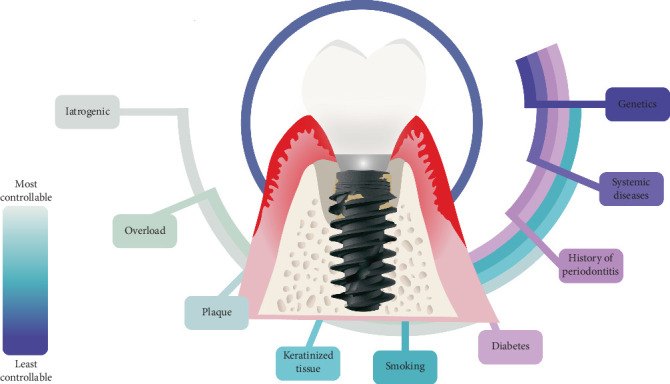
The figure illustrates the risk factors for peri-implantitis from most controllable to least controllable.

It is important to note that the studies used in the review were primarily cross-sectional or retrospective in design, which limits the ability to establish definitive causal relationships between these risk indicators and peri-implant mucositis. Further longitudinal studies are needed to better understand the role of these risk indicators in the development of peri-implant mucositis and its progression into periimplantitis (Figure 1).

## 2. Treatment

**Figure 2:**
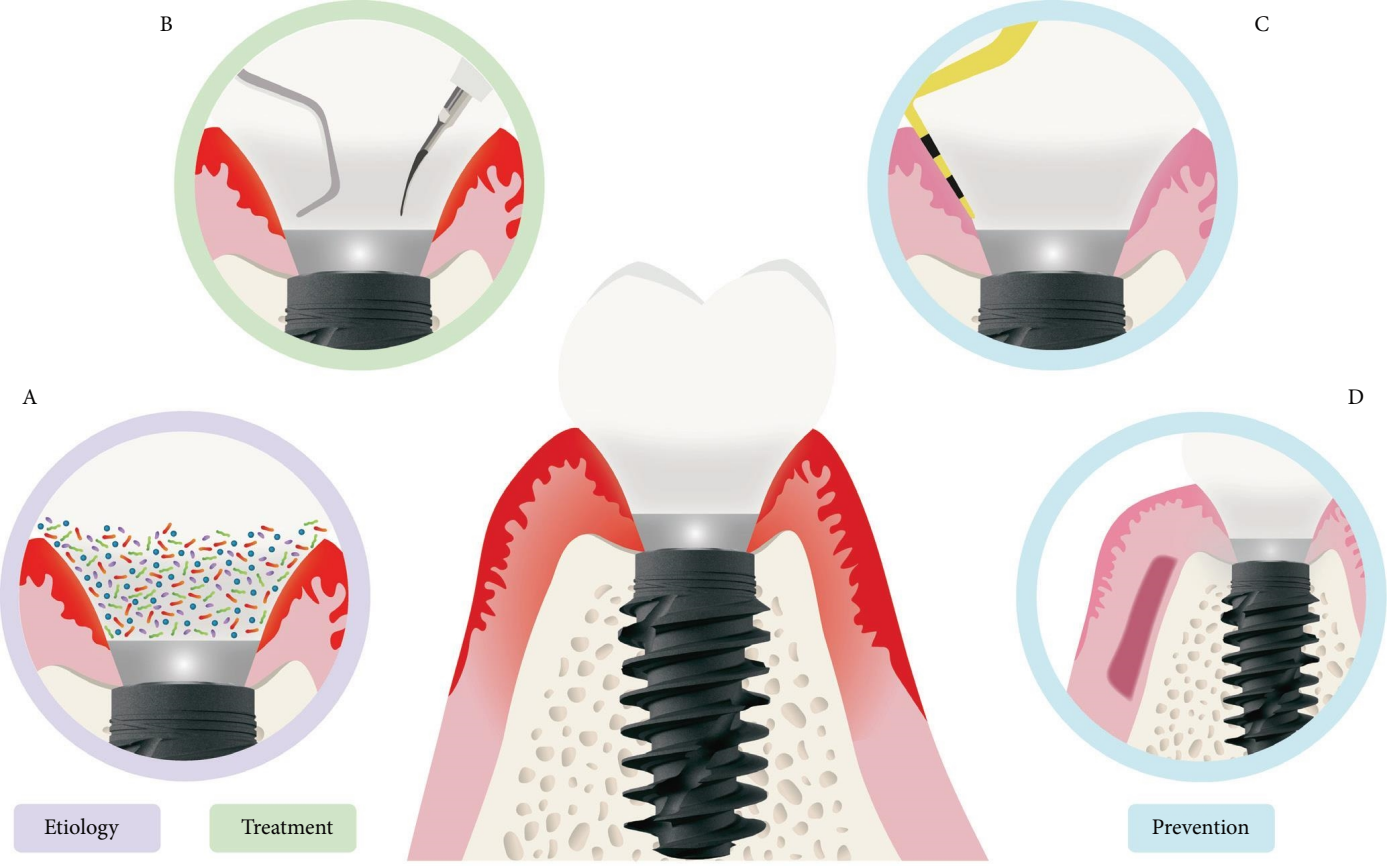
Key steps in the prevention and treatment of peri-implant mucositis. (A) Identification of the etiological cause; (B) nonsurgical mechanical debridement to remove biofilm and plaque accumulation; (C) periodical maintenance visits; (D) soft tissue grafting procedure using autogenous grafts.

The primary objective of peri-implant therapy is to eliminate contamination and detoxify the implant surface. The treatment of peri-implant diseases generally involves nonsurgical (conservative) and/or surgical approaches, depending on the severity of the disease (Figure 2). In some cases, nonsurgical therapy alone may be sufficient, while in more severe cases, a surgical intervention may be necessary.

Nonsurgical methods, such as mechanical cleaning of the implant surface using titanium or plastic curettes, ultrasonics, or air polishing, along with proper home oral hygiene techniques, are effective in managing peri-implant mucositis. Photodynamic therapy (PDT) and local antiseptic medication can also support the antimicrobial therapy.

Professional mechanical interventions, including debridement systems with curettes and ultrasonic devices, with or without the use of antimicrobials, have been shown to significantly reduce inflammation of peri-implant tissues [18]. Nevertheless, local administration of antimicrobials has not demonstrated efficacy in treating peri-implant mucositis, nor has professional irrigation of sulci with chlorhexidine (CHX) been found beneficial [15]. While all studies reviewed utilizing local antimicrobials as an adjunct to mechanical treatment showed mean improvements in BoP and probing depths, it is important to note that this therapy did not consistently resolve the lesion in all cases [15]. Therefore, professional mechanical debridement appears to be a successful treatment for peri-implant mucositis, regardless of the adjunctive use of antimicrobials [18]. However, self-administered irrigation with CHX has shown utility compared self-administration of CHX with rinsing in individuals exhibiting moderate infection signs and shallow probing depths. In such cases, irrigation proved significantly more effective in reducing inflammation signs compared to rinsing [15]. Experimental findings from different studies suggest that PDT used in conjunction with mechanical debridement is more effective in treating peri-implant conditions compared to conventional treatment alone. However, clinical investigations have produced conflicting results. Considering the discrepancies regarding the effectiveness of PDT application in treating peri-implant diseases [16]. Recently, a systematic review study found that antimicrobial PDT (aPDT) produces similar peri-implant clinical and radiographic outcomes to adjunctive antibiotic therapy when used in conjunction with mechanical debridement for treating peri-implant diseases [17].

Microbiome-based therapies, including probiotics, paraprobiotics, and postbiotics, are emerging as adjunctive treatments for peri-implant mucositis and peri-implantitis, aiming to restore microbial balance and modulate the host immune response.

Probiotics have shown mixed results as adjuncts to nonsurgical therapy. While some studies demonstrate improvements in clinical parameters such as BoP and PPD in mucositis and peri-implantitis, others report negligible microbiological effects. A recent meta-analysis concluded that probiotics may not significantly alter implant microbiota composition, but can improve clinical outcomes under specific conditions [19, 20].

Paraprobiotics and postbiotics offer promising alternatives by eliminating the challenges associated with living microorganisms; however, evidence regarding their role in the prevention and treatment of peri-implant diseases remains limited [21].

Ozone therapy exhibits powerful antimicrobial and tissue-regenerative effects by inducing lysis of bacterial cell walls and activating anti-inflammatory and tissue-repair pathways. Subgingival ozone application and ozonated water rinses have demonstrated reductions in BoP, PPD, and microbial load in peri-implant mucositis [22]. However, given the limited number of studies conducted, ozone therapy requires further evaluation as a potential approach to address reversible inflammation in peri-implant sites.

In terms of home-use oral hygiene products, mechanical plaque control combined with the use of an antiseptic can provide benefits in treating peri-implant mucositis. This approach has shown reductions in BoP, and in some cases, a decrease in the plaque index [23].

In a retrospective study conducted over a span of 7 years, Frisch et al. [24] found that patients who received supportive implant therapy had a lower incidence of peri-implant disease, specifically a reduced incidence of peri-implant mucositis (*p*=0.019). Conversely, patients who did not undergo regular maintenance had a 4.25-fold increased risk for developing peri-implantitis [24].

Barootchi et al. [25] suggest that conventional nonsurgical mechanical therapy alone can be considered the standard treatment for peri-implant mucositis, as there is currently insufficient evidence supporting the use of additional chemical or mechanical agents for clinical or microbiological improvement. Similarly, Ramanauskaite et al. [26], in a systematic review with meta-analysis, found that alternative and adjunctive measures did not provide any significant benefits in resolving peri-implant mucositis.

A systematic review conducted by Albaker et al. [27] reported that PDT or laser therapy showed improvements in clinical peri-implant inflammatory parameters such as BoP, plaque index, and PPD in the management of peri-implant mucositis. However, due to methodological heterogeneity, such as nonstandardized control groups, variations in laser parameters, and short follow-up periods, the review concluded that the findings were inconclusive. The authors stressed the need for more robust, well-designed studies with long-term follow-up and standardized comparators to further investigate the potential benefits of laser therapy in treating peri-implant mucositis.

According to Thoma et al. [28], in a systematic review and meta-analysis, soft tissue grafting procedures using autogenous grafts lead to improved peri-implant health, particularly in terms of gaining keratinized mucosa. These procedures demonstrate favorable outcomes, including reduced bleeding indices, higher marginal bone levels, and improved mucosal thickness. Additionally, the use of autogenous grafts is associated with significantly less marginal bone loss when aiming to increase mucosal thickness around dental implants.

The significance of keratinized tissue (KT) surrounding dental implants has been a subject of debate in the field. The 2006 European Association for Osseointegration (EAO) consensus conference highlighted the necessity of establishing an early and durable barrier to protect the peri-implant structures biologically. However, findings from the 2017 world workshop indicated inconclusive evidence regarding the long-term impact of KT on peri-implant tissue health. Nevertheless, there are indications that keratinized mucosa may offer benefits in terms of patient comfort during brushing, thus, facilitating plaque removal. The requirement for KT in maintaining peri-implant health has been confirmed to date, albeit with variations in defining the optimal width of KT among different studies. Hence, the absence of KT and the presence of a thin band of KT (<2 mm) should be recognized as distinct clinical conditions, despite often being grouped together in statistical analyses. Considering soft-tissue conditions as a crucial aspect of the implant treatment plan, especially in patients with shallow vestibules or thin peri-implant mucosa, is advisable from a clinical standpoint. Establishing a peri-implant soft-tissue seal, akin to a cuff encircling the implant collar, remains a paramount objective in implant dentistry to prevent peri-implant mucositis and ensure long-term implant success.

The therapeutic value of adjunctive laser therapy for the treatment of peri-implant mucositis remains uncertain due to the limited number of studies and scarcity of data available. Lin et al. [29] emphasizes the need for future clinical trials to thoroughly evaluate the potential benefits of this approach. Additional research is necessary to determine the effectiveness and applicability of laser therapy as a treatment modality for peri-implant mucositis.

### 2.1. Preventing the Progression From Peri-Implant Mucositis to Peri-Implantitis

To prevent the progression of peri-implant mucositis into peri-implantitis, a proactive and structured approach is essential. Early detection, patient education, and regular professional maintenance are critical to mitigating risk factors and ensuring disease resolution. Clinical guidelines emphasize that biofilm management, removal of prosthetic factors impeding hygiene, and supportive peri-implant therapy significantly reduce mucositis recurrence and its transition to peri-implantitis. Additionally, individualized risk assessment—considering factors such as KT width, smoking, diabetes, and prosthesis design—can guide tailored preventive interventions. Given that peri-implant mucositis remains reversible, timely nonsurgical intervention, including professional debridement and reinforcement of home care practices, should be prioritized before the disease advances to peri-implantitis

### 2.2. Practical Guidelines for Clinicians and Patients to Address Peri-Implant Mucositis

**Figure 3:**
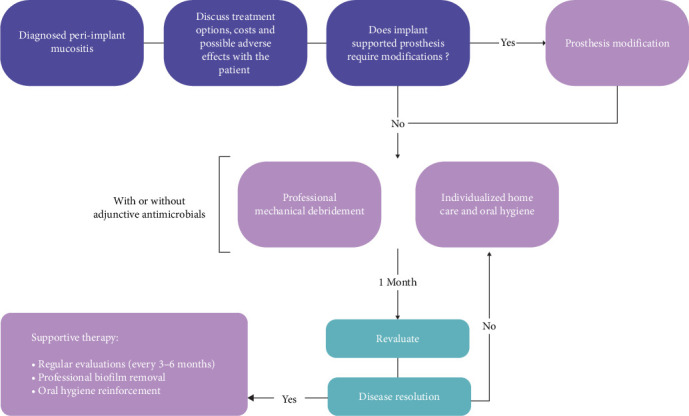
Clinical decision-making tree for treatment of peri-implant mucositis.

**Figure 4:**
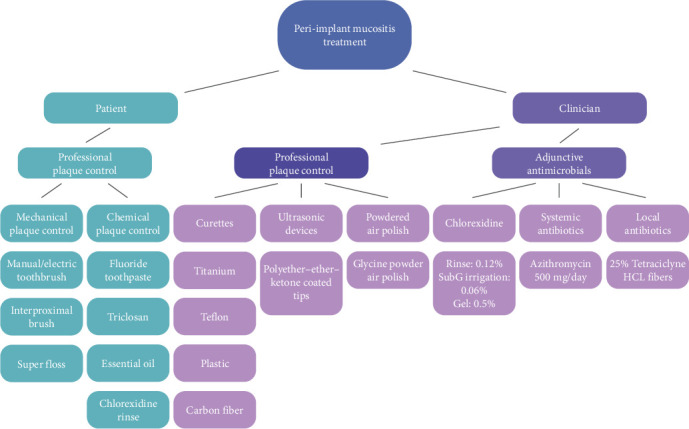
Different treatment modalities and examples of each one, divided based on patient- and clinician-related strategies.

Figure 3 depicts a structured step-by-step approach to managing cases of peri-implant mucositis. Upon diagnosing the disease (using the aforementioned criteria) and excluding peri-implantitis, the initial step involves discussing treatment options with the patient, including the rationale behind each option and potential side effects, such as peri-implant mucosal recession. In such cases, it is crucial to adequately inform patients about the potential progression to peri-implantitis and its associated consequences. Following this step, prosthesis can be assessed and in case of need for adjustment, this can be addressed. Next, the clinical- and home-care phase of the treatment is initiated. This should be performed in a collaborative approach between the clinicians and the patients to optimize the therapeutic outcomes. Reevaluation should be performed after 1 month of nonsurgical therapy at which time-point the decision is made to repeat the treatment or, in case of disease resolution (absence of BOP on gentle probing), initiate the supportive therapy.  Figure 4 further details the strategies and modalities for both clinicians and patients to utilize when addressing peri-implant mucositis. Fundamentally, the goal of treatment is to resolve inflammation in the peri-implant mucosa and to prevent the progression of this condition to peri-implantitis.

## 3. Conclusions

In summary, peri-implant mucositis poses a significant challenge in implant dentistry, affecting a substantial proportion of implant cases and carrying a risk for progression to peri-implantitis if not properly managed. The prevalence of this condition highlights the necessity for effective preventive and treatment strategies to prevent its escalation. Early intervention, risk assessment, and patient education play key roles in reducing the incidence of peri-implantitis. Nonsurgical approaches have proven effective in mitigating inflammation and controlling the disease's progression, offering promising avenues for clinical intervention. Despite this, there remains a need for further research to establish the effectiveness of adjunctive therapies, thus, underscoring the continuing effort to refine treatment approaches and improve patient outcomes. Considering that peri-implant mucositis can be a precursor to peri-implantitis, its prompt identification and proactive management are critical to the long-term health and success of dental implants. Diligence in oral hygiene maintenance and adherence to supportive care protocols are essential components of a comprehensive approach to managing peri-implant mucositis and ensuring the durability of implant treatments.

## Figures and Tables

**Table 1:** Systemic and local risk indicators for peri-implant mucositis.

Systemic risk indicators	Local risk indicators
Age	Characteristics of implant materials and surfaces
Diabetes mellitus (poorly controlled)	Dimensions of keratinized peri-implant mucosa
Radiation therapy	Lack of supportive implant therapy
Smoking	Poor oral hygiene
	Presence of excess cement
	Prostheses design

## Data Availability

The data will be provided upon reasonable request from the corresponding author.
